# Immunologic changes in the peripheral blood transcriptome of individuals with early-stage chronic Chagas cardiomyopathy: a cross-sectional study

**DOI:** 10.1016/j.lana.2025.101090

**Published:** 2025-04-17

**Authors:** Carolina Duque, Jaime So, Yagahira E. Castro-Sesquen, Kelly DeToy, Sneider A. Gutierrez Guarnizo, Fatemeh Jahanbakhsh, Edith Malaga Machaca, Monica Miranda-Schaeubinger, Indira Chakravarti, Virginia Cooper, Mary E. Schmidt, Luigi Adamo, Rachel Marcus, Kawsar R. Talaat, Robert H. Gilman, Monica R. Mugnier, the Chagas Working Group

**Affiliations:** a Department of Pathology, Johns Hopkins School of Medicine, Baltimore, MD, USA; b Department of Molecular Microbiology and Immunology, Johns Hopkins Bloomberg School of Public Health, Baltimore, MD, USA; c Department of International Health, Johns Hopkins Bloomberg School of Public Health, Baltimore, MD, USA; d Division of Cardiology, Department of Medicine, Johns Hopkins University School of Medicine, Baltimore, MD, USA; e MedStar Washington Hospital Center, Washington, D.C., USA

**Keywords:** Chagas, Cardiomyopathy, Gene expression, Immune, Biomarkers

## Abstract

**Background:**

Chagas disease, caused by the protozoan parasite *Trypanosoma cruzi,* is a neglected disease that affects approximately 6 million individuals worldwide. Of those infected, 20–30% will go on to develop chronic Chagas cardiomyopathy (CCC), and many ultimately to advanced heart failure. The mechanisms by which this progression occurs are poorly understood. In this exploratory study, we sought to provide insight into the physiologic changes associated with the development of early CCC.

**Methods:**

We used RNA sequencing to analyse the gene expression changes in the peripheral blood of six patients with Chagas disease with early structural heart disease, four patients with Chagas disease without any signs or symptoms of disease, thirteen patients without Chagas disease with early structural heart disease, and ten patients without Chagas disease or signs of heart disease. Pathway analyses and immune cell deconvolution were employed to further elucidate the biological processes underlying early CCC development.

**Findings:**

Our analysis suggests that early CCC is associated with a downregulation of various peripheral immune response genes, including changes suggestive of reduced antigen presentation and T cell activation. Notably, these genes and processes appear to be distinct from those of non-Chagas cardiomyopathies.

**Interpretation:**

This work highlights the potential importance of the immune response in early CCC, providing insight into the early pathogenesis of this disease and how it may differ from other cardiomyopathies. The changes we have identified may serve as biomarkers of early CCC and could inform future longitudinal cohort studies of markers of disease progression and strategies for the treatment of CCC in its early stages.

**Funding:**

10.13039/100000002NIH, FONDECYT, 10.13039/100006292IDSA, NSF.

**Research in context**

**Evidence before this study**

We searched MEDLINE and Google Scholar from inception to Dec 7, 2023 using the terms: (exp Chagas Disease/or exp Chagas Cardiomyopathy/) and (Blood/or “blood”.mp.) and (exp Gene expression/or “biomarker∗”.mp. or “transcriptom∗”.mp. or “differential expression”.mp. or “gene expression”.mp. or “differentially expressed”.mp.) and (limit to humans) not (review.pt.) with no language restrictions. We found 206 articles, many of which looked at biomarkers of parasitic treatment response, rather than cardiac disease progression. Of those that considered CCC disease stages, the majority focused on more advanced stages of chronic Chagas cardiomyopathy, combined early-CCC with other stages of disease, focused on markers common to most forms of cardiac damage, were very low-throughput or evaluated epigenetic alterations. We identified two studies evaluating early Chagas cardiomyopathy gene expression changes in a high-throughput manner, but neither used early non-Chagas cardiomyopathy as a comparison group.

**Added value of this study**

This exploratory study provides a high-throughput evaluation of gene expression changes in the peripheral blood of early Chagas cardiomyopathy patients, before clinical symptom onset and explores how these compare to non-Chagas cardiomyopathy patients. Our findings suggest that gene expression changes in early CCC are largely associated with dysfunction in antigen presentation and T cell activation, and that these processes are distinct from the changes associated with other early non-Chagasic cardiomyopathies.

**Implications of all the available evidence**

Our results have implications for the design of future longitudinal studies, and with further validation studies, may also inform the treatment and management of CCC. First, our study suggests that transcriptional changes can be detected in the peripheral blood of patients even at the earliest stage of CCC. Thus, the differentially expressed genes we have identified represent potential candidate biomarkers of disease progression. Since these differentially expressed genes appear to be unique to CCC, they may also serve as biomarkers of CCC-specific cardiac disease to help differentiate from other, more common causes of cardiomyopathy. In addition, our study suggests that distinct immune mechanisms underlie early CCC development, suggesting that immunomodulatory agents may prove particularly useful for the management of early CCC. New approaches for treatment are sorely needed in CCC, as anti-trypanosomal drugs are no longer effective at improving clinical outcomes once cardiac alterations have arisen. Thus, this study could guide future work towards the development of new therapeutic interventions that would be best suited for this early stage of disease.

## Introduction

Chagas disease, caused by the protozoan parasite *Trypanosoma cruzi,* is estimated to affect 6 million individuals worldwide, with an additional 70 million at risk.^1^^,^^2^ Of those infected, 20–30% will progress to chronic Chagas cardiomyopathy (CCC), a dilated cardiomyopathy resulting in symptoms of heart failure, cardiac arrhythmias, strokes, pulmonary embolisms, and/or sudden cardiac death. Together, this results in significant morbidity and an estimated 12,000 deaths per year.^1^^,^^3^^,^^4^ Currently, there is no way to predict which individuals will go on to develop CCC^5^ and the mechanisms that underly disease progression are still poorly understood.

The natural history of Chagas cardiomyopathy is highly variable. Acute infection is most commonly very mild or asymptomatic and thus the disease is rarely diagnosed in the acute stage when anti-trypanosomal drugs are most effective. While treatment in this stage is estimated to be greater than 80% effective at eliminating *T. cruzi*, it does not guarantee that a patient will not develop chronic Chagas disease.^6^, ^7^, ^8^ Patients who are not effectively treated in the acute phase will enter a chronic indeterminate phase with no signs or symptoms of disease. While most of these patients will remain asymptomatic for their entire lives, approximately one-third will gradually progress toward chronic cardiomyopathy decades after initial infection.^4^ Alterations on electrocardiogram are one of the earliest clinical indications of CCC, with the most typical being right bundle branch blocks (RBBB), left anterior fascicular blocks (LAFB), and frequent premature ventricular contractions (PVC).^9^^,^^10^ These electrical changes are a result of significant structural damage to the heart's conduction system,^11^^,^^12^ but the biologic processes contributing to this damage remain poorly understood.

Despite a dearth of research on the early processes involved in progression to CCC, evidence suggests the immune system plays an important role in the development of the cardiac damage that results in CCC. While *T. cruzi* is known to cause DNA damage, oxidative stress, and cell lysis,^13^ and thus is capable of directly damaging the heart, parasites can be difficult to detect in blood and tissues during the chronic stage of infection, thereby calling into question the extent to which the parasite alone is responsible for chronic tissue damage.^14^, ^15^, ^16^, ^17^, ^18^ In line with this, a growing body of evidence has suggested that the host immune system plays an important role in CCC pathogenesis.^19^ Histologic studies indicate that CCC, compared to non-Chagas dilated cardiomyopathy, have significantly increased CD8+ T cells, memory T cells,^18^^,^^20^^,^^21^ B cells,^20^ macrophages,^20^^,^^22^ and mast cells^21^ infiltrating cardiac tissue, all of which can contribute to tissue damage. In the peripheral blood, proinflammatory cells such as activated CD4+ T cells, NKT cells, cytotoxic NK cells,^23^ degranulating double-positive T cells,^24^ inflammatory monocytes,^25^ and cytokines such as TNF, INF-γ, and IL-6 are also increased.^26^

While together these data suggest a role for the immune system in the development of CCC, many of the observations implicating the immune system in CCC have been derived from tissues acquired during late-stage, symptomatic disease.^17^, ^18^, ^19^^,^^27^ Very few studies have focused on early CCC gene expression changes,^27^^,^^28^ and these studies utilized microarrays and qPCR, methods with technical biases that can limit differential gene expression discovery compared to more modern approaches like RNA sequencing. Moreover, these studies did not evaluate non-CCC cardiomyopathy controls, thus limiting the ability to discover biomarkers that could help differentiate CCC from other, more common, forms of cardiomyopathy. Thus, it still remains unclear what role the immune system may play in the earliest stages of CCC and whether early immunologic changes could mediate and/or signal the development of CCC in a subset of infected individuals. A better understanding of these early changes is essential given that early CCC is thought to be considerably more amenable to treatment and prevention than advanced CCC, where extensive cardiac damage has already occurred.^5^^,^^29^

In this study, we performed RNA sequencing analysis of peripheral blood samples from Chagas-positive individuals in the United States who originated from endemic regions. We focus on individuals with electrocardiographic alterations but without advanced symptomatic heart failure, with the goal of identifying very early changes that characterize the development of early-CCC and that could serve as potential biomarkers of early CCC. We identified numerous immunologic transcriptomic changes that may help differentiate early CCC from non-Chagas cardiomyopathy. Our findings suggest that the downregulation of certain immune system components may be an early indicator of CCC development.

## Methods

### Study design and participants

This study was performed under the ethics committee of the Johns Hopkins School of Public Health (protocol IRB 6713 approved Dec 16, 2015) and all individuals provided written informed consent. From 2016 to 2018, a convenience sample of individuals originally from Chagas-endemic countries were recruited from health fairs, churches, community centres, and consulates, in Virginia, Maryland and Washington D.C.^30^^,^^31^ Various geographical locations and events were chosen to minimize sampling bias. Those individuals that provided informed consent then provided a blood sample and underwent a cardiovascular evaluation consisting of a clinical history, blood pressure measurement, electrocardiogram (EKG), and echocardiogram. Self-reported demographics were also collected, including sex and country of origin.

Individuals were grouped, based on country of origin, as those from Central America (El Salvador, Guatemala, and Honduras) and those from Bolivia. We based our grouping on the general geographic distribution of *T. cruzi* discrete typing units.^32^^,^^33^

Individuals with normal EKG, normal echocardiogram, an ejection fraction (EF) greater than or equal to 50%, and no symptoms of heart failure were defined as not having cardiomyopathy. In Chagas disease, these individuals are referred to as being in the indeterminate stage of disease (IND) and are at risk of developing cardiomyopathy, although most will never develop any cardiac manifestation. The non-Chagas non-cardiomyopathy (no-CARD) individuals include those both with and without known risk factors for cardiomyopathy, especially hypertension, diabetes and obesity, but a complete clinical risk assessment was not performed. Patients with significant EKG abnormalities but normal EF ≥ 50% and no symptoms of heart failure, were defined as having an early cardiomyopathy. For Patients with Chagas disease this was termed specifically early chronic chagas cardiomyopathy (early-CCC) and for patients without Chagas disease it was termed simply early cardiomyopathy (early-CARD). The EKG abnormality inclusion criteria were: RBBB, left bundle branch block (LBBB), LAFB, left posterior fascicular block (LPFB), any atrioventricular block, bradycardia of less than 50 beats per minute, non-specific intraventricular conduction delay (NIVCD), atrial fibrillation, atrial flutter, pathologic Q waves, PVCs, ventricular tachycardia, atrial pacing or ventricular pacing. The electrocardiogram alterations are described in Supplementary Table S1, the echocardiogram ejection fractions are described in Supplementary Table S2. Co-morbidity data is described in Supplementary Table S3.

Patient serum was evaluated for anti-*T. cruzi* antibodies using the Hemagen Chagas kit (Hemagen Laboratories, Columbia, MD, USA), the Chagatest recombinant v.3.0 kit (Wiener Laboratories SAIC, Argentina), the Chagastest lysado (Wiener Laboratories SAIC, Argentina) and the IgG-TESA-blot. The IgG-TESA-blot was developed and performed using the trypomastigote excreted-secreted antigen from the *T. cruzi* Y strain.^34^^,^^35^ Individuals with positive results on two or more tests were considered seropositive for Chagas disease.^30^^,^^31^

Overall, 1571 patients were initially recruited for this study. Patients were excluded for the following reasons: they had received anti-trypanosomal medication, had incomplete Chagas tests, had a missing EKG and echo, did not have any blood collected in DNA/RNA shield, had incomplete EKG interpretations or inconsistencies between final EKG interpretation and the reported abnormalities, did not meet our EKG inclusion criteria, did not have any remaining DNA/RNA shield blood available, or if they were not from Central America or Bolivia (Supplementary Figure S1). Of the remaining 147 patients, 60 were positive for Chagas disease (10 early-CCC, 50 IND) and 87 were negative for Chagas disease (13 early-CARD, 74 no-CARD). For the early-CCC we randomly selected 7 samples for this preliminary analysis. For the non-Chagas group, we selected 10 samples due to greater variability in these patients. We then selected IND and no-CARD controls by age and sex matching samples. One of the early-CCC samples had insufficient RNA and thus was excluded, leaving 6 CCC samples.

### Sample processing

Whole blood was stored in DNA/RNA Shield (Zymo Research, Irvine, CA, USA). RNA was extracted using Quick-RNA Whole Blood Kit (Zymo Research, Irvine, CA, USA) and treated with TURBO DNAse (Thermo Fisher Scientific, Waltham, MA, USA) to remove remaining DNA contamination. Samples were then rRNA and globin depleted using the Ribo-Zero™ kit (Illumina, San Diego, CA, USA) and the GLOBINclear™ kit (Invitrogen, Waltham, MA, USA) respectively. Non-directional libraries were constructed using Novogene's proprietary kit and then loaded on the Illumina Novaseq 6000 S4 instrument per the manufacturer's instructions. The samples were sequenced using a 2 x 150 Paired-End configuration. Paired reads were adapter- and quality-trimmed using Trimmomatic (v. 0.38), aligned to the human genome GRCh38 ensembl release 84 using HiSAT2 (v. 2.1.0), and raw read counts per gene were obtained using featureCounts (v 2.0.1). All other downstream analyses were conducted in R v.4.2.2.

### Differential expression analysis and quality checking

#### Principal component analysis (PCA) and differentially expressed gene (DEG) identification

Raw counts were transformed using a variance stabilizing transformation (vst) to remove the dependence of the variance on the mean for PCA analysis using base R prcomp and visualized using ggplot2 (v.3.4.0). PCA identified considerable variance introduced by batch effect and sex (Supplementary Figure S2), so our analysis controlled for these variables by including them as covariates in the regression model.

Differential gene expression analysis between CARD vs no-CARD; and early-CCC vs IND patients was performed with DESeq2,^36^ which normalizes read counts for sequencing depth and RNA composition, estimates the dispersion for each gene and then fits a negative binomial model. Low expressed genes were removed prior to running DESeq2. These genes were defined as those for which fewer than 3 samples had 10 or more normalized counts. p-values for the differential expression of each gene were determined using the Wald test and corrected for multiple testing using the Benjamini and Hochberg (BH) adjustment. Genes with a false discovery rate (FDR) of less than 0.1 and absolute log_2_ fold change (|log_2_FC|) greater than 0.585, corresponding to a 50% change, were considered significantly differentially expressed. Given the exploratory nature of this study and the small sample size, these cutoffs were chosen to maximize discovery while still limiting falsely identified differentially expressed genes.

Heatmaps of the significant (FDR <0.1) DEGs were generated using the pheatmap package (v.1.0.12). Semi-supervised clustering of these genes (rows) was done using complete-linkage clustering of their vst transformed counts, and the distance between genes was determined by the Euclidian distance method. The code for all analyses is available at https://github.com/mugnierlab/Duque2023.

#### Gene ontology enrichment analysis

Gene ontology enrichment analysis was performed using the enrichGO and gseGO functions of clusterProfiler (v. 4.4.4).^37^^,^^38^ Both analyses were performed with a background gene set of the genes submitted to DESeq2 analysis after filtering out low-expressed genes. enrichGO applies an over-representation analysis based on a one-sided Fisher's exact test to the DEGs detected by DESeq2 analyses. gseGO applies a gene set enrichment analysis on the log2 fold change ranked list of background genes, based on the fgsea method.^37^^,^^39^ BH-adjusted p-values less than 0.05 are considered significantly enriched pathways. Gene set enrichment analyses were visualized by first using the pairwise_termsim function of clusterProfiler to calculate pairwise similarities of the enriched terms in a gene set based on the Jaccard similarity index. These were then visualized with the emapplot function using a kk layout.

#### Immune cell deconvolution

To obtain counts in transcripts per million (TPM), the previously quality trimmed fastq files were mapped and quantified with Salmon (v. 1.10.1). This used a decoy-aware human transcriptome generated by concatenating the cDNA, ncRNA, and DNA primary assembly of the GRCh38 ensembl release 109, and also corrected for fragment-level GC biases. Immune cell deconvolution of the TPM values was performed using ABSolute Immune Signal (ABIS) RNA-Seq deconvolution,^40^ CIBERSORTx with the LM22 signature-matrix and B mode batch correction,^41^ and xCell using the immunedeconv (v.2.1.0) package.^42^ Groups were compared using the Wilcox-rank sum test with BH adjustment for multiple comparisons.

#### Demographic statistical analyses

Descriptive and inferential statistics were conducted using R v.4.2.2. Normality of data was assessed using visual examination and the Shapiro–Wilk test. Fisher's exact test or student t-test were used where appropriate.

#### Role of the funding source

The funding sources for this work had no role in the study design, data collection and analysis, interpretation of results, or writing of this article.

## Results

### Transcriptional changes in peripheral blood are associated with early CCC

**Fig. 1:**
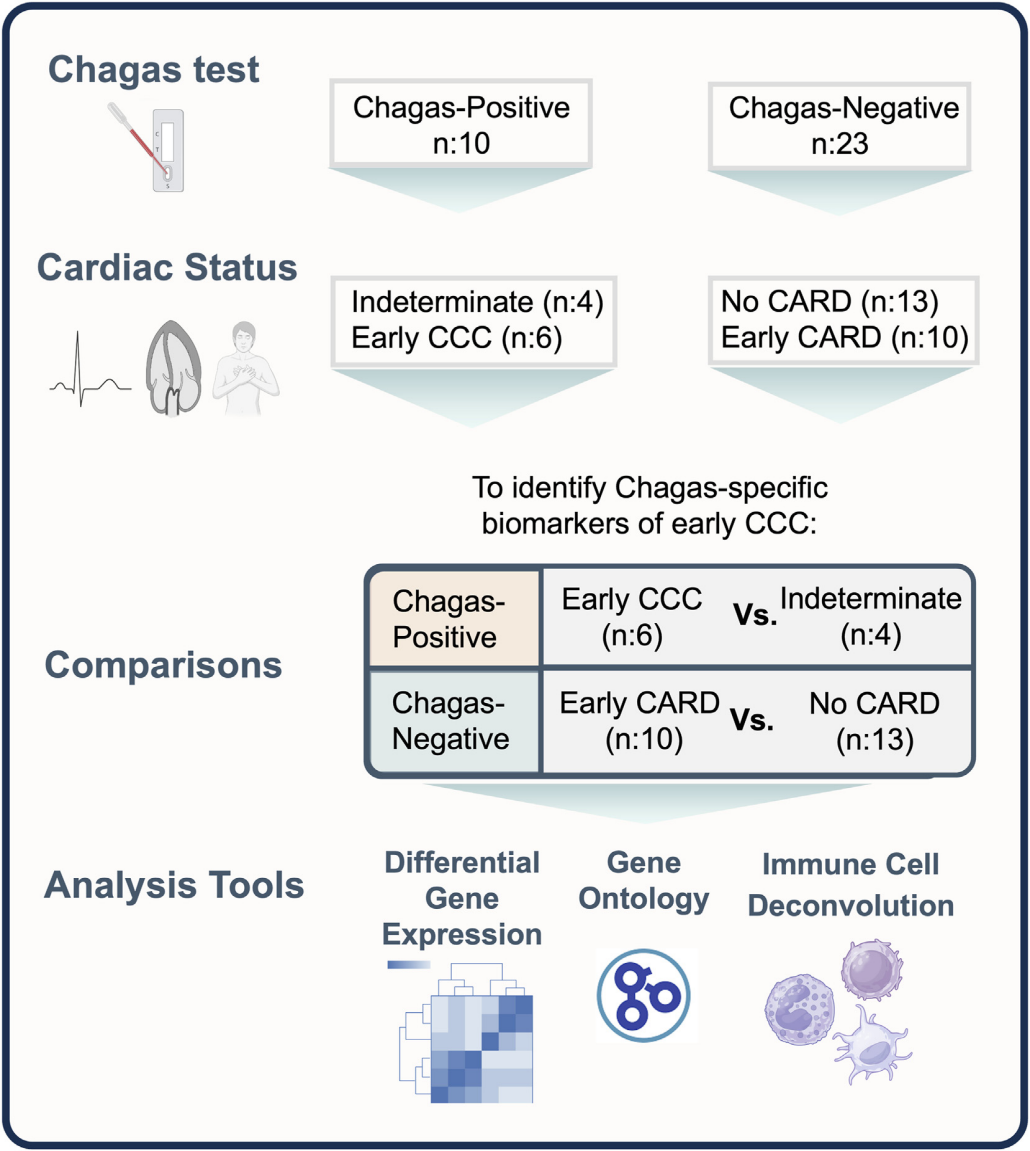
**Study design schematic**. 10 Chagas-positive patients were randomly selected, and 23 Chagas-negative patients were matched by age, sex, region of origin, and cardiac disease stage. These patients were stratified by Chagas status to compare cardiac patients with early cardiomyopathy (non-Chagas early-CARD or early-CCC) (abnormal EKG, normal echocardiography, no symptoms of heart failure) to those with no signs or symptoms of cardiomyopathy using differential gene expression, gene ontology and immune cell deconvolution. Abbreviations: CARD, cardiomyopathy; CCC, chronic Chagas cardiomyopathy. Image created with BioRender.com.

We sought to investigate whether gene expression changes in peripheral blood were associated with early heart disease and whether these signatures were different for Chagas-positive and Chagas-negative patients. We, therefore, compared individuals with no signs or symptoms of cardiac disease to those with early cardiac abnormalities detectable by EKG in both Chagas-seropositive and seronegative individuals matched by age and sex (Fig. 1, Table 1). Given the small sample size, we controlled for but did not stratify by sex in our RNAseq regression models.

**Table 1:** Study participant demographic data.

	Chagas-positive			Chagas-negative		
	No evidence of cardiomyopathy (IND)	Early cardiomyopathy (Early-CCC)	p-value	No evidence of cardiomyopathy (No-CARD)	Early cardiomyopathy (Early-CARD)	p-value
Number	4	6		13	10	
Mean Age (sd)	51.85 (15.19)	44.36 (4.59)	0.28	47.22 (10.05)	48.68 (12.25)	0.76
Female (%)	3 (75)	2 (33)	0.52	9 (69)	5 (50)	0.42
Bolivians (%)	4 (100)	4 (67)	0.47	9 (69)	6 (60)	0.69
Central Americans (%)	0 (0)	2 (33)		4 (31)	4 (40)	

p-value determined by Fisher's exact test or student t-test. Sd-standard deviation for each group. % - percentage for each group.

**Fig. 2:**
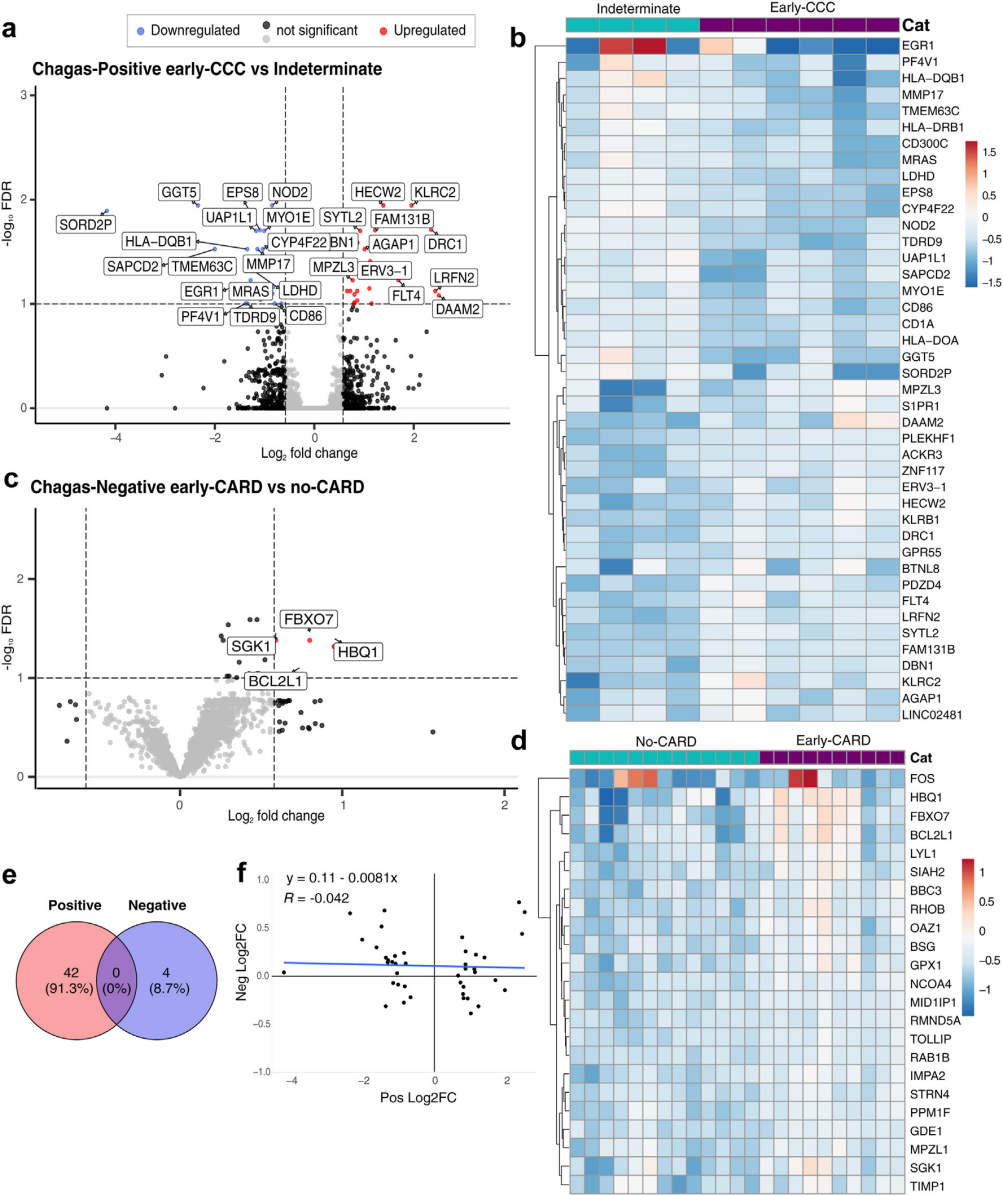
**Distinct gene expression changes characterize early CCC. (a,c)** Volcano plot of patients with early cardiomyopathy (CCC or CARD) vs those with no evidence of cardiomyopathy (IND or non-CARD) for **a)** chagas seropositive and **c)** seronegative individuals. Upregulated DEGs (FDR <0.1 and log_2_FC > 0.585) are shown in red, downregulated DEGs (FDR <0.1 and log_2_FC < −0.585) are in blue, genes with FDR <0.1 or |log_2_FC| > 0.585 but not both are in black, and non-significant DEGs are in grey. (**b,d)** Heatmap of relative vst-transformed values for the most significant DEGs with FDR <0.1 in chagas **b)** seropositive and **d)** seronegative individuals. Color represents the sample's difference from the mean for that gene. **e)** Venn diagram of significant DEGs of early cardiomyopathy vs non-cardiomyopathy for chagas seropositive and seronegative individuals. **f)** scatter plot of log2FC of seropositive DEGs vs the log2FC of these same genes in seronegative individuals with Pearson correlation test. Trendline shown in blue. Abbreviations: CARD, cardiomyopathy; CCC, chronic Chagas cardiomyopathy; IND, indeterminate.

In Chagas-positive patients, we identified 42 significant (FDR <0.1, |log_2_FC| > 0.58) DEGs. Of these 21 were upregulated and 21 downregulated in early-CCC compared to indeterminate stage individuals (Fig. 2a and b, Supplementry Table S4). Various downregulated genes reflect immunologic processes including antigen presentation based on multiple major histocompatibility complex (MHC) classes (HLA-DRB1, HLA-DQB1, HLA-DOA), and CD1A^43^; T-cell activation (CD86)^44^; myeloid activation (CD300C)^45^; and activation of various immune cell types (nucleotide-binding oligomerization domain containing 2 (NOD2))^46^ Upregulated immune genes reflect processes associated with both inhibitory and stimulatory signals in T cells and NK cells (killer cell lectin-like receptor B1 (KLRB1))^47^, ^48^, ^49^ and stimulatory roles in NK cells (killer cell lectin-like receptor C2 (KLRC2)).^50^

Conversely, for individuals without Chagas, we identified only 4 significant (FDR <0.1, |log_2_FC| > 0.585) DEGs, all of which were upregulated in early-CARD compared to patients with no evidence of cardiomyopathy (Fig. 2c and d, Supplementary Table S5). This smaller number of DEGs may be due to greater variability of aetiologies and EKG alterations in seronegative individuals. Nevertheless, our data suggest that the underlying gene expression changes associated with early heart disease (EKG-detectable cardiomyopathy vs no evidence of cardiomyopathy) might be distinct in these Chagas-negative controls compared to Chagas-positive patients, as none of the significant DEGs intersect with those of Chagas-positive individuals (Fig. 2e). To ensure that this lack of intersection is not the result of small effect sizes, we also performed a correlation analysis that showed no clear association (R = −0.042, p = 0.79) between the effect sizes of the log_2_FC of the Chagas-positive DEGs and the log_2_FC of these same genes in Chagas-negative patients (Fig. 2f). Together these data could indicate that dynamic changes in immune components accompany the development of early-CCC and that these changes may be unique to early-CCC pathogenesis but need to be confirmed in future studies with larger sample sizes.

### Early-CCC is characterized by reduced antigen presentation and immune cell activation

**Fig. 3:**
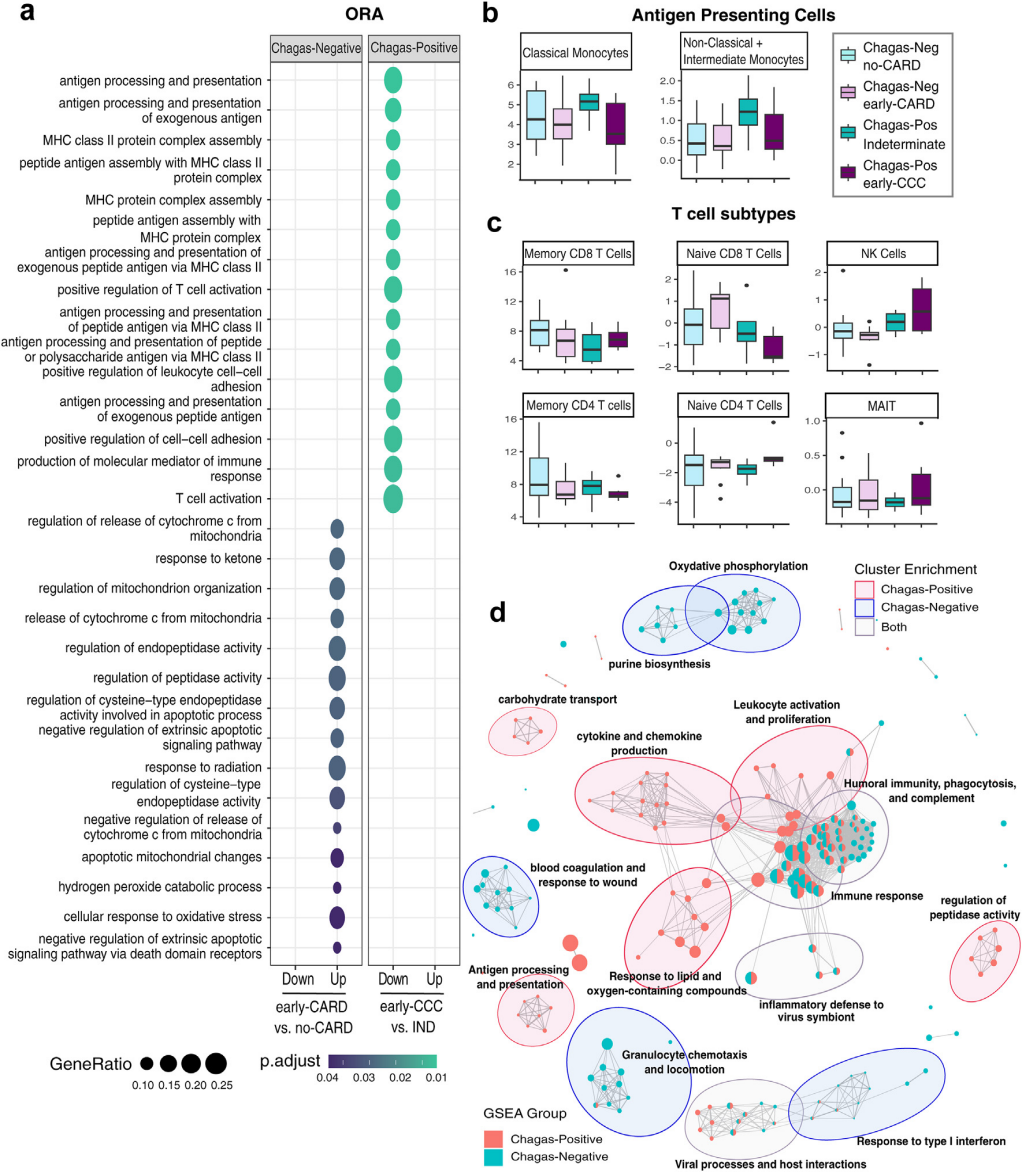
**Early-CCC may be associated with reduced antigen presentation and T cell activation. a)** top 15 over-represented (ORA) GO biologic process (BP) terms (FDR <0.05) for downregulated (Down) and upregulated (Up) DEGs for early cardiomyopathy vs non-cardiomyopathy patients that are Chagas seronegative (Neg) or seropositive (Pos). Circle size represents number of genes in the given pathway, color represents the FDR. **b)** box plot of antigen presenting cell deconvolution from bulk RNAseq data using ABsolute Immune Signal (ABIS) **c)** box plot of T cell deconvolution from bulk RNAseq data using ABIS **d)** enrichment map of gene set enriched analysis (GSEA) GO BP for seronegative and seropositive patients with grey lines connecting overlapping gene sets, and showing 15 clusters with >2 biologic processes. Node size represents number of genes enriched in the pathway and line length represents pairwise similarity. Cluster color represents the comparison with predominant enrichment with red reflecting predominantly pathways enriched for Chagas positive, blue for Chagas negative, and grey when there is an near even enrichment for both groups. Abbreviations: MAIT, Mucosal-associated invariant T cell; NK, Natural Killer; Pos, Chagas seropositive; Neg, Chagas seronegative; CARD, cardiomyopathy; IND, indeterminate.

Having found various transcriptomic changes associated with early-CCC, we sought to explore the biologic processes underlying these changes. Using GO analysis, we found that in the Chagas group, no pathways were overrepresented in the upregulated genes, but 129 biologic processes were significantly over-represented (FDR <0.05) in the downregulated genes. These pathways were largely associated with antigen presentation and T cell function and were mainly driven by the DEGs: CD86, early growth response 1 (EGR1), HLA-DRB1, HLA-DQB1, HLA-DOA, NOD2, and CD1A (Fig. 3a, Supplementary Table S6). Immune cell deconvolution also shows a trend toward a possible reduction in classical and non-classical monocytes (antigen presenting cells) in Chagas early-CCC compared to the indeterminate stage, though this finding did not reach statistical significance (Fig. 3b, Supplementary Figure S2). Conversely, T cell subsets, including mucosal-associated invariant T cells, CD4 naïve T cells, and CD8 memory T cells, as well as NK cells, do not show any consistent change in early-CCC (Fig. 3b, Supplementary Figure S3). This suggests that the alterations in T cell activation are unlikely to be due to a reduction in T cell populations but could potentially be due to fewer antigen presenting cells or a reduction in their activation signalling. These trends were not seen in Chagas-negative controls (Fig. 3b and c). Furthermore, when analysing individuals without Chagas disease with early cardiomyopathy vs those with no evidence of cardiomyopathy, GO analysis showed no overlapping pathways with those of the Chagas disease group. These 24 biologic processes that were enriched in the non-Chagas disease early cardiomyopathy group (compared to the non-cardiomyopathy group), were largely related to apoptotic mitochondrial processes and oxidative stress pathways (Fig. 3a, Supplementary Table S6). These pathways have been strongly associated with ischemic, diabetic, and other causes of cardiomyopathy.^51^ This finding further highlights that CCC may have a distinct pathophysiology from at least some other forms of cardiomyopathy.

To further verify the finding of CCC-specific immune alterations, we performed a gene set enrichment analysis of the complete fold change ranked gene list. This allows us to identify pathways that have gene expression changes that are be too small or noisy to be considered differentially expressed, but that are consistently occurring in the same coordinated direction for that given pathway. Based on this analysis, we identify 111 significantly (FDR <0.05) enriched biologic processes for the Chagas positive group, and 119 for the Chagas negative group (Fig. 3d, Supplementary Table S7), of which 46 are overlapping. While many of the overlapping processes are related to general aspects of immunity, there are notable immunological differences between the Chagas and non-Chagas groups. Chagas-positive individuals are enriched for pathways relating to antigen presentation, leukocyte activation and proliferation, cytokine and chemokine production, and response to oxygen and lipid-containing compounds, while Chagas-negative individuals are enriched for granulocyte chemotaxis, and response to type-I interferons. For non-immunologic processes, Chagas-positive individuals are enriched for pathways relating to the transport of carbohydrates, amino acids, and cholesterol, and regulation of peptidase activity. Conversely, Chagas-negative patients are enriched for coagulation, oxidative phosphorylation, and purine biosynthesis.

## Discussion

In this study, we have used peripheral blood samples from individuals with and without Chagas disease in the early stages of cardiomyopathy to gain insight into the physiologic processes that may underly the development of CCC. Our findings suggest that, despite having a chronic *T. cruzi* infection, patients with chronic Chagas disease developing early signs of CCC may show immunologic alterations suggestive of a dysregulated immune response in the peripheral blood, rather than a hyperactive one. Notably, these changes appear to be evident even before patients become clinically symptomatic and were not seen in patients with non-Chagas cardiomyopathies. This suggests that the mechanism by which CCC develops may be distinct from that of certain other non-Chagas cardiomyopathies. These results further highlight the potentially important role that the immune system could play in CCC pathogenesis, while also providing evidence for the feasibility of identifying patients that may develop early-CCC before they actually develop any clinical symptoms.

Our results shed light on the potential immune mechanisms at play during the earliest stage of CCC. We found a notable downregulation of immune-associated genes in the peripheral blood of patients with Chagas disease with electrocardiographic manifestations compared to those in the indeterminate stage without any signs or symptoms of disease. Specifically, we observe a pattern of gene expression that strongly indicates a downregulation in antigen presentation and T cell activation in the peripheral blood. T cell activation is generally considered to depend on three signals 1) antigen presentation 2) co-stimulatory signalling and 3) cytokine signalling, and in this study, we found a downregulation of genes corresponding to all three of these signals. First, various antigen-presenting genes were downregulated including various MHC molecules and CD1a.^52^ For signal 2, the co-stimulatory protein CD86 was downregulated, and for signal 3, EGR1, an important transcription factor for various T cell stimulatory cytokines,^53^ was reduced. Consistent with our findings, Ferreira et al. also showed reduced HLA-DPB1 expression in early-CCC compared to PCR-negative indeterminate stage patients,^27^ and Gomez-Olarte et al. showed that cells infected with *T. cruzi* have reduced expression of co-stimulatory molecules such as CD86^54^ and antigen-presenting MHC proteins *in vitro*.^55^^,^^56^ In line with this, our immune cell deconvolution analysis suggests that although T cell numbers are not decreased, certain antigen presenting cells, which are required for T cell activation, may be decreased in the peripheral blood of early CCC patients. Together these data point toward a model in which T cell function could be compromised in the peripheral blood of patients that develop early CCC, possibly due to a reduction in antigen-presenting cells or a dysfunction in their stimulatory signalling to T cells.

Notably, in contrast with our findings, the current paradigm in the field is that CCC pathogenesis is largely a pro-inflammatory process.^19^^,^^23^, ^24^, ^25^, ^26^ This logically stems from the infectious nature of Chagas disease, where a strong immune response against *T. cruzi* could result in cardiac tissue damage. Most prior work supporting this paradigm has focused on severe CCC or has not distinguished between different stages of CCC for analysis. Such experimental designs, while informative, are likely to mask transient changes specific to earlier stages of disease. Nevertheless, there is a growing body of CCC research showing indicators of downregulated immune responses, similar to what we have observed in this study, such as downregulated innate immune markers, increased T cell exhaustion marker expression, and increased T regulatory cells.^19^^,^^57^, ^58^, ^59^, ^60^ T cell responses are also critical for controlling *T. cruzi* parasitaemia.^61^^,^^62^ Thus, early T cell dysfunction may allow for greater parasite persistence and continuous low-level parasite-mediated cardiac damage. Once sufficient additional cardiac damage has occurred, immune responses may resurge due to the production of damage-associated molecular patterns and autoantigens,^63^ helping explain the largely pro-inflammatory findings seen in studies of more advanced CCC.^19^^,^^23^, ^24^, ^25^, ^26^ Thus, the complete picture of CCC pathogenesis is likely more nuanced, and potentially dynamic, than the current paradigm suggests. Our study is notable in its focus on very early-stage CCC and highlights the value of stratifying patients by disease stage for comparison.

In addition, while there are limitations to the analysis of peripheral blood in CCC—most notably that it may not always reflect the processes occurring at the site of cardiac damage^64^, ^65^, ^66^—there is value in understanding the changes in this easily accessible tissue. Our results here suggest that it is possible to find changes in gene expression in the peripheral blood of early CCC patients. This suggests that it may be feasible to identify blood biomarkers of early-CCC development even before the clinical EKG manifestations of disease. It would be highly valuable to be able to risk-stratify the individuals that are most likely to develop CCC and that would most benefit from treatment, because it is currently impossible to identify the 20–30% of patients who will progress to CCC. Indeed, anti-trypanosomal drugs are currently recommended for all patients younger than 50 in the indeterminate stage of disease,^67^ even though these drugs involve long treatment regimens and can be associated with notable side effects.^5^^,^^68^ Peripheral blood biomarkers would thus be valuable for informing treatment decisions in CCC and helping to identify those individuals in need of specialized treatment and closer follow-up. It is possible that some of the differentially expressed genes identified in this study could be used in this way, but future longitudinal studies will be essential for validating these markers of disease progression.

This study also suggests that the transcriptional signature and immunologic processes associated with early CCC appear to be largely distinctive of Chagas disease. If, as our results tentatively suggest, the underlying mechanisms mediating cardiac damage are distinct between Chagas and certain forms of non-Chagas heart disease, then the clinical management for these diseases should likely also be different. In particular, our findings suggest that immunomodulatory agents may prove useful for the management of early CCC. This may be particularly beneficial since anti-trypanosomal drugs are no longer effective at improving clinical outcomes once cardiac alterations have arisen.^5^

This study provides important insight into the potential processes underlying the development of early CCC, but it is subject to certain limitations. First, despite using early heart failure stages to understand early disease pathogenesis, this study is still limited by its cross-sectional design. This study may thus help guide future longitudinal analyses that will be essential to understand the processes that precede disease progression and to understand immune dynamics throughout infection. In addition, our relatively small sample size, with large person-to-person variation, may have limited our ability to detect more subtle gene expression changes between groups. We used a FDR cutoff of <0.1 to mitigate this limitation to optimize the discovery of candidate genes in this exploratory analysis, but this also may result in more false positive results. A larger sample size will also allow for a more thorough evaluation of sex, race, ethnicity, comorbidities, and *T. cruzi* strain differences in progression to early CCC. This will also allow us to better match patients on a variety of criteria and will allow us to better compare CCC progression to a wider variety of non-Chagas cardiomyopathies. In addition, a validation cohort is needed to validate our findings and the ability of these potential biomarkers to assign individuals into early CCC, indeterminate or non-Chagas cardiomyopathy. Furthermore, while gene expression is important in understanding the pathogenesis of CCC, we did not evaluate whether this reflects changes at the protein level; this will be necessary to fully understand the phenotypic changes in early-CCC. Future studies will thus be needed to address these limitations.

Overall, we have identified potential immunologic markers of early chronic Chagas cardiomyopathy that could help shed light on the pathogenesis of this disease. This work suggests that measurable changes in peripheral blood gene expression can be detected in CCC patients even before they become clinically symptomatic and that the mechanisms mediating the development of early-Chagas cardiomyopathy may be distinct from non-Chagas cardiomyopathies. These insights provide a path towards using peripheral biomarkers to identify patients most likely to progress to early-CCC, and also towards new immunologic treatment approaches for this early, and perhaps more treatable stage of disease.

## Contributors

RHG, KRT, and YEC designed the study. RHG, YEC, and MS obtained funding for the study. YEC, MM, IC, VC, KD, and FJ collected and processed samples for the study. EMM, YEC, and FJ performed the Chagas diagnostic tests. RM performed the electrocardiograms, the echocardiograms and their interpretation for the study. MS performed clinical questionnaires. JS performed the sample matching, the RNA extractions, and the quality checks for all samples. CD performed the data analysis. CD and SAGG performed the data visualization. CD and MRM drafted the manuscript. LA and MRM helped interpret the results. All authors reviewed and edited the manuscript. CD, YEC, KD, RM, and MRM accessed and verified the underlying data of the study. All authors had full access to all the data in the study and accept responsibility for the decision to submit for publication.

## Data sharing statement

The raw RNA-seq data from this study have been deposited in the Gene Expression Omnibus (GEO) database (https://www.ncbi.nlm.nih.gov/geo/, GSE244827). The code for generating the analysis and figures in this paper is available at https://github.com/mugnierlab/Duque2023.

## Declaration of interests

R.H.G. reports nonfinancial support from InBios International Inc., in the form of free Chagas rapid tests, during the conduct of the study and outside the submitted work. LA is co-founder of i-Cordis, LLC, a start-up company focused on the development of immunomodulatory molecules for the treatment of heart failure. LA is also a consultant for Kiniksa Pharmaceuticals and Novo Nordisk.

## Appendix A Supplementary data

Supplementary Figures and Table

STable4

STable5

STable6

STable7

Spanish abstract
