# Supplementary material for: Immunologic changes in the peripheral blood transcriptome of individuals with early-stage chronic Chagas cardiomyopathy: a cross-sectional study
Source: Lancet Reg Health Am. 2025 Apr 17;45:101090. doi: 10.1016/j.lana.2025.101090 (PMC12033964; doi:10.1016/j.lana.2025.101090)
Supplement: Supplementary Figures and Table [file mmc1.pdf]

Supplementary Appendix

Table of Contents

Supplementary Figure 1 .... **2**

Supplementary Figure 2 .... **3**

Supplementary table 1 .... **5**

Supplementary table 2 .... **6**

Supplementary table 3 .... **7**

Supplementary table 4 .... **8**

Supplementary table 5 .... **8**

Supplemental Table 6 .... **8**

Supplemental Table 7 .... **8**

Supplementary Figure 1
Trial profile

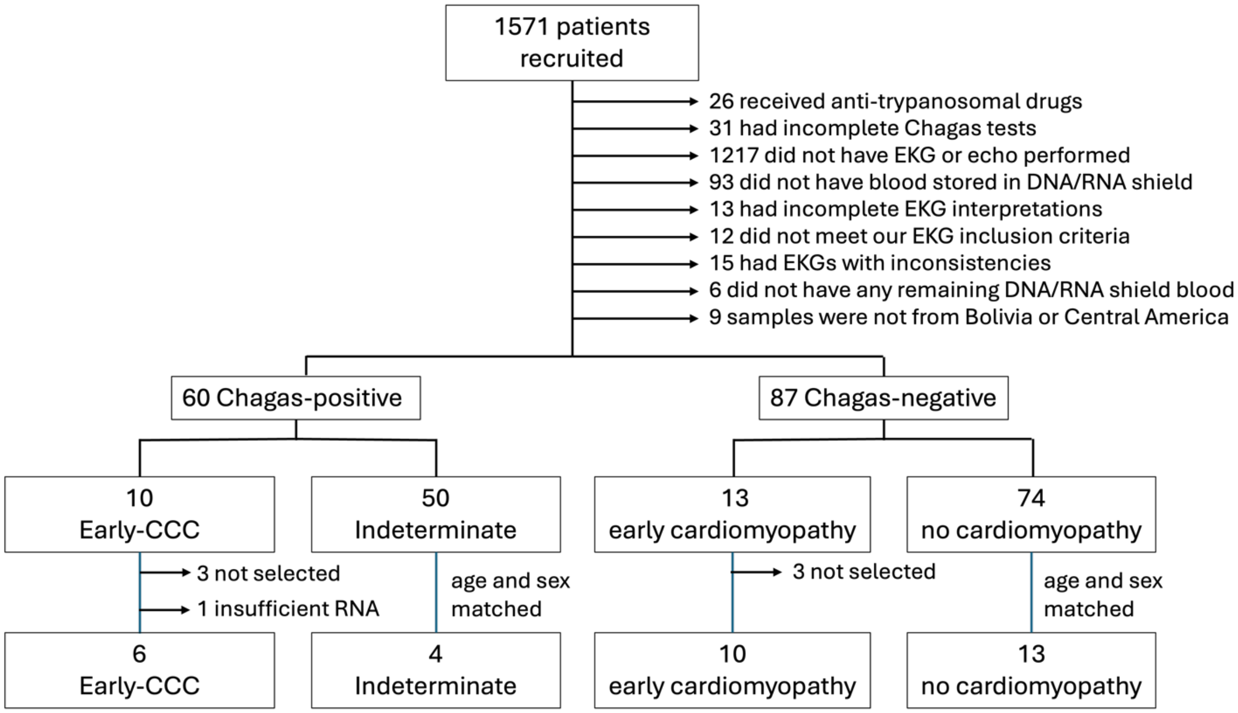

Supplementary Figure 2

Sex and sequencing batch contribute to significant variance in the data. Principal component analysis (PCA) plot of vst normalized gene counts. Principal component (PC) 1 vs PC2 colored by **a)** sequencing batch and **b)** sex. Pairwise plots of PC1 – PC5 with a density diagram along the diagonal and colored by **c)** age category, **d)** Chagas serostatus, **e)** heart failure stage, **f)** region of origin. F – female, M – Male, Neg – Chagas negative, Pos- Chagas Positive, Bol – Bolivian, CentAm – Central American.

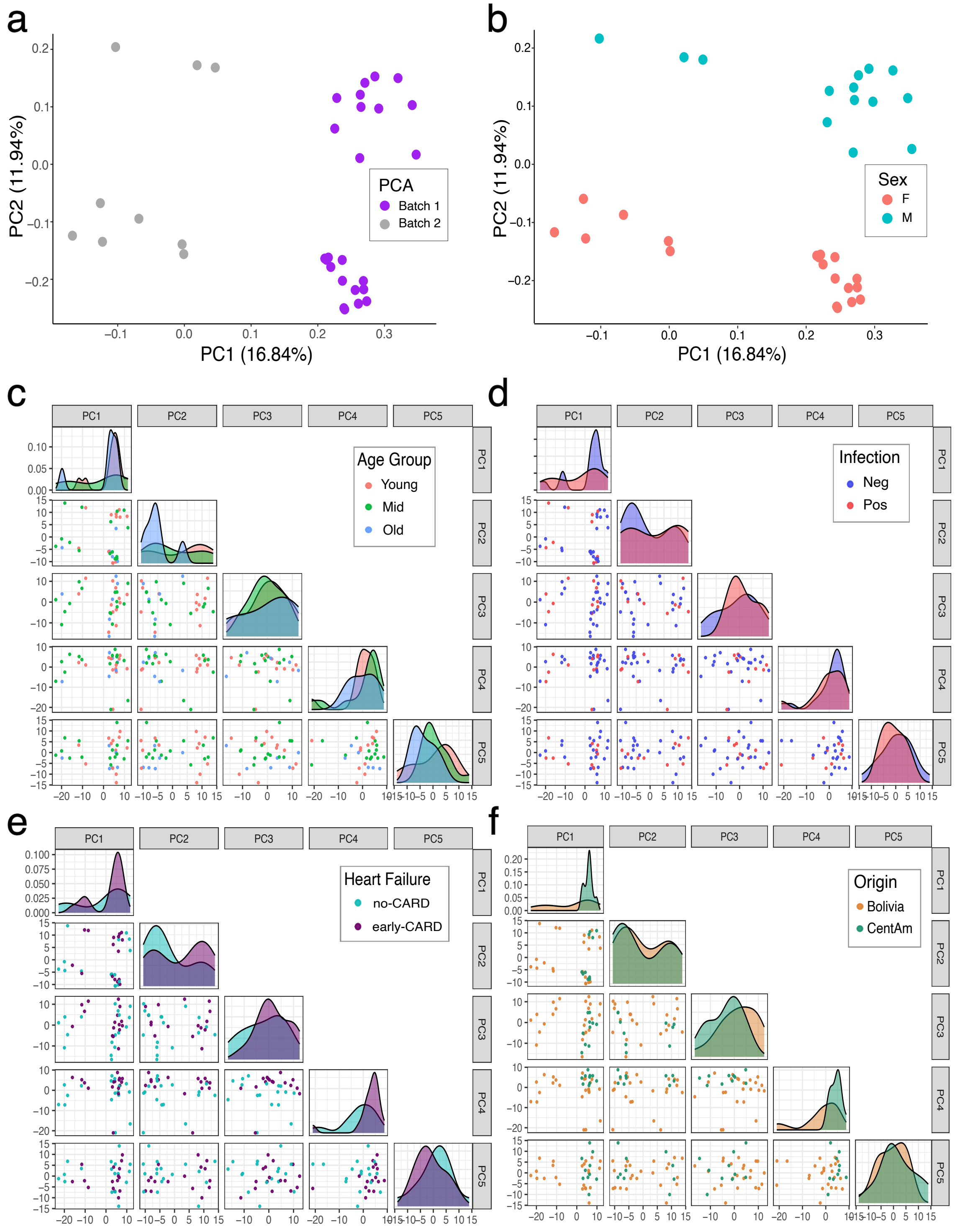

Supplementary Figure 3

Antigen presenting cell and T cell subset changes are generally consistent across deconvolution methods. box plot of B cell deconvolution of bulk RNAseq data for Chagas-negative no-CARD, Chagas-negative early-CARD, Chagas-positive no-CARD and Chagas-positive early-CCC patients. Deconvolution was performed using ABsolute Immune Signal (ABIS), CIBSERSORTx and xCell for a) B cells, b) myeloid dendtritic cells, c) monocytes d) CD8 T cells, e) Memory CD4 T cells f) Naïve CD4 T cells, and g) Natural Killer cells. Abbreviations: C – Classical, NC + I – Non-Classical and Intermediate, CM – Central memory, Mem – memory, NK – Natural Killer, Pos – Chagas seropositive, Neg – Chagas seronegative, CARD- cardiomyopathy, CCC- chronic Chagas cardiomyopathy

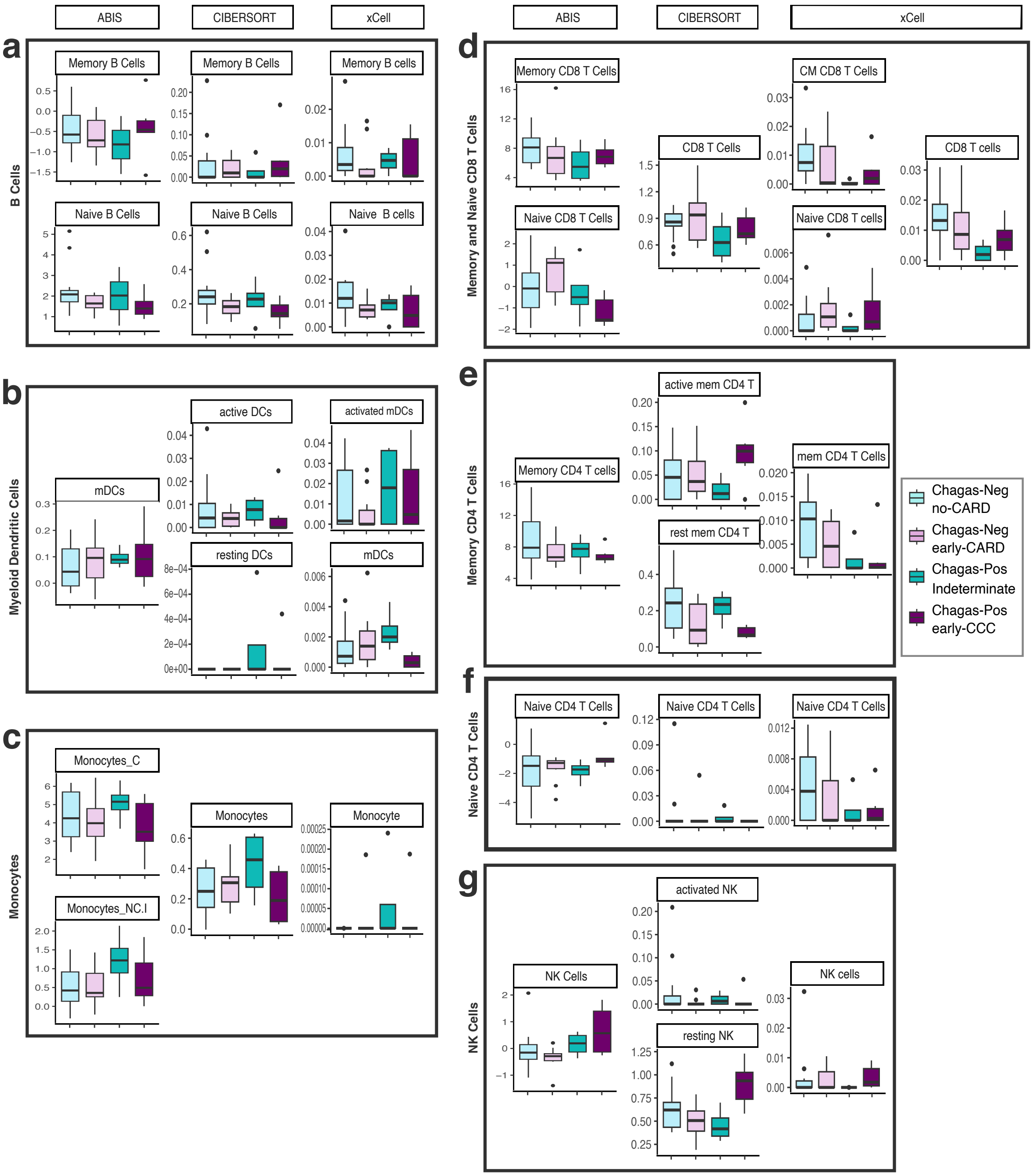

Supplementary table 1

Electrocardiogram (EKG) findings for early-cardiomyopathy patients by serostatus. P-value determined by Fisher's exact test

	**Chagas-Positive**	**Chagas-Negative**	p-value
n	10	6	
Bradycardia (%)	2 (20.0)	2 (33.3)	0.604
LPFB (%)	3 (30.0)	1 (16.7)	1
LAFB (%)	3 (30.0)	2 (33.3)	1
T wave abnormality (%)	1 (10.0)	0 (0.0)	1
PVC (%)	1 (10.0)	0 (0.0)	1
First degree AV block (%)	2 (20.0)	1 (16.7)	1
LVH (%)	1 (10.0)	1 (16.7)	1
IVCD (%)	3 (30.0)	2 (33.3)	1
RBBB (%)	1 (10.0)	3 (50.0)	0.118
Low voltage (%)	1 (10.0)	0 (0.0)	1

Supplementary table 2

Ejection fraction by serostatus and cardiac disease stage. P-value determined by t-test.

	Chagas-Negative		Chagas-Positive		
	**No evidence of cardiomyopathy (No-CARD)**	**Early cardiomyopathy (Early-CARD)**	**No evidence of cardiomyopathy (Indeterminate)**	**Early Chronic Chagas Cardiomyopathy (Early-CCC)**	p
n	13	10	4	6	
consensus_EF (mean (SD))	63.56 (5.92)	64.67 (9.24)	58.25 (3.95)	68.33 (4.16)	0.266

Supplementary table 3

Comorbidities by serostatus and cardiac disease stage. P-value determined by Fisher's exact test or t-test. Note, 4 patients have missing data for hypertension, 12 have missing data for diabetes, and 9 have missing BMI.

		**Chagas-Negative**		**Chagas-Positive**		
		**No evidence of cardiomyopathy (No-CARD)**	**Early cardiomyopathy (Early-CARD)**	**No evidence of cardiomyopathy (Indeterminate)**	**Early Chronic Chagas Cardiomyopathy (Early-CCC)**	p
n		13	10	4	6	
Hypertension (%)	Elevated_BP	2 (18.2)	0 (0.0)	0 (0.0)	1 (20.0)	
	Hypertension	5 (45.5)	2 (22.2)	2 (50.0)	2 (40.0)	
	Normal	4 (36.4)	7 (77.8)	2 (50.0)	2 (40.0)	0.537
Diabetes (%)	Diabetes	1 (14.3)	0 (0.0)	0 (0.0)	0 (0.0)	
	Normal	6 (85.7)	7 (100.0)	3 (100.0)	4 (100.0)	1
BMI (mean (SD))		29.69 (4.09)	30.32 (4.01)	33.37 (3.44)	28.30 (7.22)	0.545

Supplementary table 4

Differentially expressed gene list with false discovery rate (FDR) less than 0.1 for Chagas-positive early-CCC compared to indeterminate stage patients without any evidence of cardiomyopathy showing the ensembl stable gene id, the base mean, the log_2 fold change (log2FC), the standard error of the log2FoldChange estimate (lfcSE), the wald statistic (stat), the pvalue, the false discovery rate (FDR) and the HUGO gene nomenclature external gene name. Genes were considered significantly differentially expressed when the log_2 fold change is greater than 0.585 or less than -0.585 and the FDR is less than 0.1.

(see attached excel)

Supplementary table 5

Differentially expressed gene list with false discovery rate (FDR) less than 0.1 for Chagas-negative early-cardiomyopathy patients compared to patients without any evidence of cardiomyopathy showing the ensembl stable gene id, the base mean, the log_2 fold change (log2FC), the standard error of the log2FoldChange estimate (lfcSE), the wald statistic (stat), the pvalue, the false discovery rate (FDR) and the HUGO gene nomenclature external gene name. Genes were considered significantly differentially expressed when the log_2 fold change is greater than 0.585 or less than -0.585 and the FDR is less than 0.1.

(see attached excel)

Supplemental Table 6

Significantly enriched (FDR < 0.05) gene ontology biologic process terms from over representation analysis for downregulated (Down) and upregulated (Up) differentially expressed genes for both Chagas-positive (pos) and Chagas-negative (neg) early-cardiomyopathy (early-CCC or early-CARD) vs no-cardiomyopathy (IND or no-CARD) comparisons.

(see attached excel)

Supplemental Table 7

Significantly enriched (FDR < 0.05) gene ontology biologic process terms from gene set enrichment analysis for downregulated (Down) and upregulated (Up) differentially expressed genes for both Chagas-positive (pos) and Chagas-negative (neg) early-cardiomyopathy vs no-cardiomyopathy comparisons.

(see attached excel)
