# Supplementary material for: Immunologic changes in the peripheral blood transcriptome of individuals with early-stage chronic Chagas cardiomyopathy: a cross-sectional study
Source: Lancet Reg Health Am. 2025 Apr 17;45:101090. doi: 10.1016/j.lana.2025.101090 (PMC12033964; doi:10.1016/j.lana.2025.101090)
Supplement: Spanish abstract [file mmc6.docx]

Editor´s disclaimer: *This translation in Spanish was submitted by the authors and we reproduce it as supplied. It has not been peer reviewed. Our editorial processes have only been applied to the original abstract in English, which should serve as reference for this manuscript*

**Introducción**

La enfermedad de Chagas, causada por el parásito *Trypanosoma cruzi,* es una enfermedad desatendida que afecta a aproximadamente 6 millones de personas. Se estima que entre el 20 y el 30% de los pacientes infectados desarrollarán cardiomiopatía chagásica crónica (CCC) e insuficiencia cardíaca avanzada. Los mecanismos que conducen a la progresión de CCC son poco conocidos. En este estudio exploratorio, buscamos proporcionar evidencia sobre los cambios fisiológicos asociados con el desarrollo temprano de CCC.

**Métodos**

Utilizamos el secuenciamiento de ARN para analizar los cambios en la expresión génica detectables en la sangre periférica de seis pacientes con Chagas con enfermedad cardíaca estructural temprana, cuatro pacientes con Chagas sin ningún signo o síntoma de la enfermedad, trece pacientes sin Chagas con enfermedad cardíaca estructural temprana y diez pacientes sin Chagas sin signos o síntomas de enfermedad cardíaca. Analizamos vías génicas y utilizamos la deconvulación de células inmunológicas para dilucidar los procesos biológicos que subyacen al desarrollo temprano de CCC.

**Resultados**

Nuestro análisis sugiere que la CCC temprana se asocia con una disminución en la expresión de varios genes de respuesta inmunológica periférica, incluyendo cambios que sugieren una reducción de la presentación de antígenos y la activación de células T. Notablemente, nuestro análisis resalta genes y procesos biológicos únicos de CCC cuando se compara con otras miocardiopatías tempranas no-chagásicas.

**Interpretación**

Este trabajo resalta la importancia potencial de la respuesta inmunológica en el desarrollo temprano de CCC, proporcionando información sobre la patogénesis temprana de esta enfermedad y de sus diferencias respecto a otras miocardiopatías. Los cambios que hemos identificado pueden servir como biomarcadores de CCC temprana y podrán informar futuros estudios de cohortes longitudinales sobre marcadores de progresión de la enfermedad y estrategias para el tratamiento oportuno de CCC.

**Financiación**

NIH, FONDECYT, IDSA, NSF
